# Closing the implementation gap: the Joint Action on Cardiovascular Diseases and Diabetes (JACARDI) as a proof of-concept for Europe’s Safe Hearts Plan

**DOI:** 10.3389/ijph.2026.1609906

**Published:** 2026-06-15

**Authors:** Benedetta Armocida, Beatrice Formenti, Albert Aszalos, Hector Bueno, Ewelina Chawłowska, Hanna Elonheimo, Mounia El Yamani, Irati Erreguerena, Sinikka Kyto, Matilde Leonardi, Jaana Lindström, Agnes Makai, Bernardino Morillo, Luigi Palmieri, Roberta Papa, Markku Peltonen, Helena Safadi, Natalia Skogberg, Anna Tarhanchuk, Hanna Tolonen, Edwin Wouters, Jelka Zaletel, Katarzyna Zukowska, Graziano Onder

**Affiliations:** 1 Istituto Superiore di Sanità, Rome, Italy; 2 Fondazione Policlinico Gemelli, Rome, Italy; 3 Gottsegen National Cardiovascular Center, Budapest, Hungary; 4 Centro Nacional de Investigaciones Cardiovasculares (CNIC), Madrid, Spain; 5 Hospital Universitario 12 de Octubre and Instituto de Investigación Sanitaria Hospital 12 de Octubre (imas12), Madrid, Spain; 6 Fundación para la Investigación Biomédica del Hospital Universitario 12 de Octubre (FIBH12O), Madrid, Spain; 7 Poznan University of Medical Sciences, Poznan, Poland; 8 Finnish Institute for Health and Welfare, Helsinki, Finland; 9 Santé Publique France, Saint Maurice, France; 10 Network for Research on Chronicity, Primary Care, and Health Promotion (RICAPPS), Biosistemak Institute for Health System Research, Bilbao, Spain; 11 Fondazione IRCCS Istituto Neurologico Carlo Besta, Milan, Italy; 12 University of Eastern Finland, Kuopio, Finland; 13 FundeSalud - Foundation for the Training and Research of Health Professionals in Extremadura, Merida, Spain; 14 Agenzia Regionale Sanitaria, Regione Marche, Ancona, Italy; 15 Semmelweis University, Faculty of Health and Public Administration, Health Services Management Training Centre, Budapest, Hungary; 16 Public Health Center of the Minitry of Health of Ukraine, Kiev, Ukraine; 17 University of Antwerp, Antwerp, Belgium; 18 National Institute of Public Health Slovenia, Ljubljana, Slovenia; 19 Università Cattolica del Sacro Cuore, Rome, Italy

**Keywords:** cardiovascular disease, diabetes, joint action, public health, safe hearts plan

## Introduction

Cardiovascular diseases (CVDs) remain the leading cause of premature mortality and disability across European Union (EU), imposing a substantial and inequitably distributed burden on health systems and economies [1]. The recently launched EU Safe Hearts Plan (SHP) reflects renewed political commitment to cardiovascular health, establishing a coordinated agenda across prevention, early detection, and integrated cardiometabolic care [2]. Yet the central challenge lies not in designing strategic frameworks but in translating them into consistent, equitable implementation across heterogeneous health systems. CVD risk is shaped by intersecting biological, behavioural, and social determinants that evolve across a lifetime. Bridging the strategy-to-implementation gap requires operational models that act on this full life course complexity.

The Joint Action on Cardiovascular Diseases and Diabetes (JACARDI), a collaborative initiative involving 81 public health institutions across 21 European countries, offers a valuable proof-of-concept for how this translation can be achieved [3]. This commentary argues that JACARDI’s portfolio of over 140 pilots, spanning prevention, screening, data infrastructure, integrated care, self-management, and workplace health, constitutes life course-oriented evidence base with long-term impact, from which the SHP’s implementation architecture can directly draw.

## A life course framework for cardiometabolic action: building cardiovascular health from childhood

A defining feature of JACARDI is that it addresses CVDs and diabetes within a unified cardiometabolic framework. Shared biological pathways, overlapping risk factor profiles, and frequent clinical co-occurrence demand that prevention, detection, and management strategies address both conditions simultaneously [4], offering a model for integrating other comorbidities in future frameworks. JACARDI maps this integrated imperative into a life course continuum, recognising that cardiometabolic risk is a continuous trajectory shaped by cumulative exposures, life transitions, and the quality of care available at each stage. This framing operationalises the SHP’s three pillars, population prevention, early detection, and coordinated care, as interdependent rather than sequential. Indeed, the foundations of cardiometabolic health are laid in childhood and adolescence, when risk behaviours, biological risk factors, and health literacy competences are most amenable to modification. JACARDI pilots in this phase include school-based health literacy and lifestyle programmes engaging children aged 6–12 in Aragón, Spain, combining nutrition and physical activity promotion through a co-design model involving students, families, educators, and primary care professionals. Co-designed youth health literacy workshops in the Czech Republic and the Basque Country extend this approach to adolescents, integrating broader social and care environment. Latvia’s paired familial hypercholesterolaemia screening pilots targeting children aged 5–7 and young adults demonstrate how systematic hereditary risk detection can be operationalised with scalable referral pathways.

## Detection and the prevention-care interface: reaching adults at risk

As cumulative risk exposure manifests as detectable biological change in adulthood, the prevention-to-care interface becomes a critical juncture in the cardiometabolic continuum. JACARDI has developed a Short Guide for Screening Individuals at Increased Risk of CVD and type 2 diabetes mellitus [5], consolidating the conceptual landscape of risk identification and emphasising that detection generates health benefit only when explicitly linked to follow-up and care. Pilot evidence illustrates a range of approaches: Hungary’s mobile health clinic units deliver CVD and diabetes risk assessment in geographically underserved communities; Ireland generates evidence for a natriuretic peptide-based heart failure screening model with direct clinical guideline implications; and Belgium operationalises the detection-to-intervention continuum by linking online risk assessment tools to coordinated lifestyle support programmes. The shared lesson is that effective screening is an organisational and social process requiring design that reaches populations in everyday environments and links identification to actionable follow-up.

## Integrated care and self-management: living with cardiometabolic conditions

For patients, the life course framework shifts from prevention to sustained, person-centred management. Fragmentation between hospital, primary, and community care remains a structural weakness across European health systems [6]. JACARDI adopts a co-design approach, engaging patients, providers, and local authorities to ensure context-appropriate integrated care models. JACARDI pilots include value-based, multidisciplinary heart failure care pathways co-developed with patients and caregivers, incorporating sex/gender inequalities in the Basque Country; integrated post-stroke pathways embedding structured follow-up in Aragón; telemedicine-supported management of heart failure and diabetes in Italy’s Autonomous Province of Trento; and embedding comprehensive, structured health education into post acute myocardial infarction (AMI) rehabilitation care in Slovenia. Complementing these models, digital self-management tools extend therapeutic support into patients' everyday lives: a co-designed mobile application to optimise adherence to post-AMI secondary prevention in Madrid; and digital and telemedicine-enabled care models for diabetes and heart failure, aimed at strengthening self-management, continuity of care, and integration between health services in Marche Region, Italy. These pilots demonstrate that effective integrated care requires both clinical coordination and patient self-management support and that digital tools can extend both when co-designed with users and connected to health system infrastructure. JACARDI also addresses the life course, including the employment sector. Specific workplace pilots in Italy, Finland, Lithuania, and Poland focus on prevention and reintegration to work.

## Data infrastructure and equity: transversal requirements

Two imperatives cut across the entire life course continuum: robust data infrastructure and structural equity. JACARDI has addressed both as foundational investments. Its conceptual data framework, Common Indicator Model, and OMOP common data model v5.4 standardisation guidelines provide a shared technical architecture for cardiometabolic monitoring that can also be used to monitor variations and inequities by sex, age, and socioeconomic position as a default feature, aligned with the European Health Data Space (EHDS) federated model [7, 8]. Pilots in Italy, Finland, Iceland, and Latvia are translating this architecture into practice through harmonised diabetes and CVD registries, national data repositories, and patient-reported outcomes integrated into quality registers. Spain’s EUROCARDIAB pilot extends this further, developing a federated pan-European platform with an embedded policy simulator projecting the population-level impact of prevention strategies, a direct analytical tool for SHP accountability.

On equity, JACARDI has embedded a transversal Equity and Diversity Framework, grounded in intersectionality theory and the social determinants of health, as a methodological requirement throughout pilot design and implementation [9]. Its four principles (critical reflection, co-design, context and data, inclusive communication) shape who defines the problem, whose data are considered, whose voices are heard, who is included and who has access, ensuring that no one is left behind. All JACARDI projects are provided capacity strengthening and consultations in integrating equity in their activities: health literacy interventions for post-partum women and women from economically disadvantaged areas in France and Italy; blue-collar workers and drivers reached through occupational health channels in Poland; involvement of managers of enterprises in Italy to promote inclusiveness of persons with non-communicable diseases; migrant communities co-designing prevention in Portugal, Finland, and the Czech Republic; healthcare professionals engaged in anti-racism training to deliver inclusive care. Minimum equity data standards for cardiometabolic registries, defining essential sociodemographic variables, remain a key frontier: systems that cannot detect inequities cannot address them. Strengthening sex- and gender-disaggregated data is critical for advancing equity in cardiovascular health, as shown by pilots exploring how gender-related socioeconomic inequalities shape outcomes in Spain and France.

### Conclusion

The SHP provides a comprehensive strategic framework for addressing the cardiometabolic burden across Europe. JACARDI’s contribution is to demonstrate, through over 140 pilots, that this framework can be operationalised as a coherent life course continuum. Three structural lessons emerge: i) the life course is an operational framework for cardiometabolic action; ii) embedding an intersectional equity framework, operationalised through co-design and interoperable, equity-stratified data systems, is essential to reach marginalised populations and ensure accountability in implementation; iii) digital innovation complements but does not substitute for health system reform. As JACARDI’s evidence continues to consolidate, its translation into SHP implementation designed for long term impact represents an effective route from political commitment to population health impact.

## Group members of JACARDI Implementation Board

Jose Carlos Arévalo, Jessica Barbieri, Tommaso Bertossi, Ludovica Borsoi, Anja Brunec, Fatima Sanchez Cabo, Alice Colombo, Cecilia Damiano, Katrien Danhieux, Sonia Dias, Chiara Donfrancesco, Inmaculada Fernandez, Enrica Finotto, Arianna Fornari, Ane Fullando, Claudia Giacomozzi, Cinzia Giammarchi, Andrea Guajardo Villar, Yhasmine Hamu, Judit Kolcza, Agnieszka Lipiak, Paula Maillo, Domitilla Marconi, Alessia Marcassoli, Maria J. Marques, Angelica Mazzilli, Sara Mellano, Tomi Mäki-Opas, Mariia Murasheva, Laura Musta, Maria Nousiainen, Denis Opresnik, Eeva Rantala, Mieke Rijken, Carla Rognoni, Rodrigo Ruiz-Martinez, Franco Sassi, Marco Silano, Uliana Snidevych, Teresa Spadea, Nora Strommer, Melinda Szogi, Giulia Trucco, Paivi Valve, Gergely Varga, Julia Wolska.
